# The Surgery of the Past

**Published:** 1888-07-21

**Authors:** 


### THE SURGERY OF THE PAST.

Of all the surgical triumphs of modern times, none are regarded with more pride than those operations on the skull which so often save a patient from insanity or a long agony of death in life. "Thanks to anæsthetics, instruments of extreme fineness, and advancing science," say the operators, "we dare venture on operations our forefathers never contemplated," and they never suspect that their forefathers of the long-past Stone Age not only contemplated but succeeded in the dangerous operation of trephining. Who the brave surgeon was who first attempted an operation of such magnitude we do not know, nor who was the not less brave patient who first submitted to it; but the practice goes back to such old-world times that some of the learned say that the legend of the birth of Pallas, springing fully-armed from the brain of Jove, is a science-myth relating to the operation of trephining.

Leaving myths, however, we come to facts that indicate that this operation was known, was even popular, among semi-savage nations in prehistoric times. In the year 1868 M. Prunières discovered, in a dolmen near Aiginères, a skull in which a large hole had been cut. He thought it was one of those grim drinking-cups from which the heroes were supposed to quaff the mead in Valhalla, and which those who aspired to reach Valhalla practised using on earth with the skulls of their dead enemies; but as more and more of these cups were found, Dr. Paul Broca, a man of science, began to doubt the accuracy of the explanation. He studied the skulls carefully, and came to the conclusion that where the mutilation was smooth it represented a cicatrised wound, healed before death, while the jagged edges of other parts showed where pieces of the skull had been cut after death by friends or others who wished to obtain some portion of the old wound by way of relic or amulet. The way in which Dr. Broca traced back his theory from the allusions to such operations in mediæval books, through traditions that were rife before printing was invented, and supplemented the knowledge thus gained by information as to the surgery of savage nations in modern times, cannot be detailed here in full; but he brought very satisfactory evidence to support his case. A work published by Jehan Taxil in 1603, spoke of the practice of removing the dura mater for the relief (?) of infantile convulsions, then confounded with epilepsy. It is probable, therefore, that the first patients who underwent trephining were infants. How many of these hapless babies perished under the knife, or rather flint, can never be known; but those who survived were not unreasonably looked upon with a certain reverence, and thence by the natural logic of the savage mind a fragment of their mutilated skulls acquired a superstitious value after their death. As such an operation indulged in for any reason or none might occasionally leave the victim's mind weak, the connexion—so devoutly believed in by the ignorant—between madness and superior sanctity would immediately be established.

The theory of the operation seems to have been this: Epilepsy and kindred disorders were mysterious diseases, not directly traceable to the action of heat or cold or any obvious physical conditions. Being thus inexplicable by ordinary causes, they were supposed to be caused by evil spirits. The hole in the head was intended to provide an exit for these tormenting demons, and it is surmised that the tonsure, which is the sign of the priesthood of more than one creed, is a mild survival of the prehistoric belief that the man with the hole in his head was one from whom evil spirits had departed. Later, when types had been evolved from facts, the removal of hair from the crown implied the resolve to expel the devil and take up a sacred vocation.

This operation is still practised in the South Sea Islands, where a piece of broken glass is the favourite instrument since the introduction of that European luxury has caused the original flint or shark's tooth to be cast aside. A circular cut is made, and the bone of the skull is then scraped away within the limits thus traced out. In a child the operation may be finished in five minutes; in an adult it takes at least an hour. After it is over a piece of cocoa-nut shell ground smooth and to the desired shape is placed over the wound to protect the tissues thus exposed. That any mortal should meekly sit down and submit to such an agonizing operation seems incredible; yet it appears to be a fashionable one, so popular that hardly any self-respecting male among the inhabitants is without a hole in his cranium. Moreover, it is a point of honour for the patient not to show that he suffers. A similar practice was common among the Kabyles of Algeria, and one skull decorated with a square hole above the forehead has been found in a Peruvian grave. As the edges of the wound were not completly healed, Dr. Broca, to whom the skull was submitted, surmised that the patient had died not long after the operation. Let us hope that in this case the trephining was a grave scientific experiment, the result of which would suffice to discourage any further rash attempt at cranial surgery.

